# Complete Genome Sequence of *Streptococcus agalactiae* Serotype III, Multilocus Sequence Type 283 Strain SG-M1

**DOI:** 10.1128/genomeA.01188-15

**Published:** 2015-10-22

**Authors:** Kurosh S. Mehershahi, Li Yang Hsu, Tse Hsien Koh, Swaine L. Chen

**Affiliations:** a National University of Singapore, Singapore; b Saw Swee Hock School of Public Health, Singapore; c Singapore Infectious Diseases Initiative, Singapore; d Singapore General Hospital, Singapore; e Genome Institute of Singapore, Singapore

## Abstract

*Streptococcus agalactiae* (group B *Streptococcus*) is a common commensal strain in the human gastrointestinal tract that can also cause invasive disease in humans and other animals. We report here the complete genome sequence of *S. agalactiae* SG-M1, a serotype III, multilocus sequence type 283 strain, isolated from a Singaporean patient suffering from meningitis.

## GENOME ANNOUNCEMENT

*Streptococcus agalactiae* (group B *Streptococcus*, or GBS) is a Gram-positive bacterium commonly found as a commensal in the gastrointestinal and genitourinary tract of up to 40% of humans (1). *S. agalactiae* can cause pregnancy-associated infections, which can lead to invasive neonatal disease (bacteremia, pneumonia, and meningitis) after delivery; invasive disease (bacteremia, meningitis, and soft tissue infections) in all age groups; and urinary tract infections (2, 3). Furthermore, GBS is an important veterinary pathogen in several animals, including cows (mastitis) (4) and fish (meningoencephalitis) (5). GBS has been classified by serotype (Ia, Ib, II to IX) (6). Strain SG-M1 is a serotype III clinical isolate of GBS, isolated from a patient in Singapore suffering from meningitis; this isolate was collected as part of an outbreak investigation in Singapore associated with the consumption of raw fish (Barkham et al., unpublished data), in accordance with Singapore ethics regulations for exemption from IRB review. By multilocus sequence typing (http://pubmlst.org/sagalactiae), SG-M1 was determined to be a multilocus sequence type 283 strain (ST283); previously, ST283 strains were isolated from a meningitis patient in Hong Kong (7) and aquatic animals, the latter representing a potential zoonotic source (8).

SG-M1 was grown overnight in brain heart infusion broth at 37°C with shaking. The bacterium was collected by centrifugation and lysed in a buffer containing 20 mM Tris-Cl (pH 8.0), 2-mM sodium EDTA, 1.2% Triton X-100, and 20 mg/mL lysozyme from chicken egg white (MP Biomedicals cat. no. 100831) at 37°C for 45 min. Genomic DNA was then extracted using a Qiagen QIAamp DNA minikit and sheared to a size of approximately 10 kbp using g-Tube (Covaris). A 10-kb SMRTBell library was prepared for sequencing according to the protocols recommended by Pacific Biosciences, loaded with a MagBead-bound library protocol, and sequenced using P5-C3 chemistry on the PacBio RS II instrument (Pacific Biosciences) with a 240-min movie time on two SMRTCells. *De novo* assembly was performed with the Hierarchical Genome Assembly Process (HGAP3) in the SMRT Analysis suite version 2.3 using default parameters (9). In total, there were 62,129 reads and 740,417,316 nucleotides that passed filtering, representing an approximate coverage of 260× (based on the final assembly) and a preassembly mean read length of 11,917 bp.

*S. agalactiae* SG-M1 harbors a single chromosome of 2,116,810 bp with a G+C content of 35.5%; no plasmids were found. Annotations of the CI5 genome and plasmid were performed using the NCBI Prokaryotic Genome Annotation Pipeline (PGAAP) (10). SG-M1 contains 2,037 protein coding sequences, as well as 7 rRNA operons and 81 tRNA genes. The finished genome sequence of *S. agalactiae* SG-M1 will aid in further understanding the causative basis for invasive disease caused by GBS.

### Nucleotide sequence accession number.

The complete sequence of the *S. agalactiae* strain SG-M1 chromosome has been submitted to GenBank under the accession number CP012419.
