# Erratum for Mehershahi et al., Complete Genome Sequence of *Streptococcus agalactiae* Serotype III, Multilocus Sequence Type 283 Strain SG-M1

**DOI:** 10.1128/genomeA.01585-15

**Published:** 2015-12-31

**Authors:** Kurosh S. Mehershahi, Li Yang Hsu, Tse Hsien Koh, Swaine L. Chen

**Affiliations:** a National University of Singapore, Singapore; b Saw Swee Hock School of Public Health, Singapore; c Singapore Infectious Diseases Initiative, Singapore; d Singapore General Hospital, Singapore; e Genome Institute of Singapore, Singapore

## ERRATUM

Volume 3, no. 5, e01188-15. 2015. Page 1, column 2: Lines 5–7 should read as follows. “. . . Annotation of the SG-M1 genome was performed using the NCBI Prokaryotic Genome Annotation Pipeline (PGAAP) . . . .”

